# Compound screening in human airway basal cells identifies Wnt pathway activators as potential pro-regenerative therapies

**DOI:** 10.1242/jcs.263487

**Published:** 2025-04-14

**Authors:** Yuki Ishii, Jessica C. Orr, Marie-Belle El Mdawar, Denise R. Bairros de Pilger, David R. Pearce, Kyren A. Lazarus, Rebecca E. Graham, Marko Z. Nikolić, Robin Ketteler, Neil O. Carragher, Sam M. Janes, Robert E. Hynds

**Affiliations:** ^1^ Lungs for Living Research Centre, UCL Respiratory, University College London, London WC1E 6JF, UK; ^2^ Epithelial Cell Biology in ENT Research Group, Developmental Biology and Cancer Department, UCL Great Ormond Street Institute of Child Health, University College London, London WC1N 1DZ, UK; ^3^ MRC Laboratory for Molecular Cell Biology, University College London, London WC1E 6BT, UK; ^4^ UCL Cancer Institute, University College London, London WC1N 6DD, UK; ^5^ Edinburgh Cancer Research, Institute of Genetics and Cancer, University of Edinburgh, Western General Hospital, Edinburgh EH4 2XU, UK; ^6^ Cancer Research UK Scotland Centre, University of Edinburgh, Edinburgh EH4 2XU, UK

**Keywords:** Lung regeneration, Bronchial epithelial cells, Cell proliferation, Compound screening, Respiratory biology, β-catenin

## Abstract

Regeneration of the airway epithelium restores barrier function and mucociliary clearance following lung injury and infection. The mechanisms regulating the proliferation and differentiation of tissue-resident airway basal stem cells remain incompletely understood. To identify compounds that promote human airway basal cell proliferation, we performed phenotype-based compound screening of 1429 compounds (from the ENZO and Prestwick Chemical libraries) in 384-well format using primary cells transduced with lentiviral luciferase. A total of 17 pro-proliferative compounds were validated in independent donor cell cultures, including the antiretroviral therapy agent abacavir and several Wnt signalling pathway-activating compounds. The effects of compounds on proliferation were further explored in colony formation and 3D organoid assays. Structurally and functionally related compounds that more potently induced Wnt pathway activation were investigated. One such compound, 1-azakenpaullone, induced Wnt target gene activation and basal cell proliferation in mice. Our results demonstrate the pro-proliferative effect of small-molecule Wnt pathway activators on airway basal cells. These findings contribute to the rationale to develop novel approaches to modulate Wnt signalling during airway epithelial repair.

## Teaser

**Highlighted Article:** A high-throughput screen of 1429 compounds in primary human airway epithelial cells was used to identify that Wnt pathway-activating compounds increase cell proliferation.

## INTRODUCTION

Human airways are lined by a pseudostratified epithelium that acts as a physical barrier and enacts mucociliary clearance to remove inhaled particles and pathogens (Bustamante-Marin and Ostrowski, 2017). Maintaining these functions during homeostasis and after injury is crucial for lung health. Tissue-resident basal stem cells support this process by self-renewing and differentiating to replenish airway epithelial cell lineages (Basil et al., 2020; Rock et al., 2009).

Airway epithelial repair is integral to respiratory health, and is required during influenza, respiratory syncytial virus (RSV) and rhinovirus infections, where epithelial desquamation occurs (Johnson et al., 2007; Taubenberger and Morens, 2008; Turner et al., 1982; Walsh et al., 1961). Chronic lung diseases also involve basal cell dysfunction, with cells from donors with asthma (Kicic et al., 2010; Stevens et al., 2008) or chronic obstructive pulmonary disease (Ghosh et al., 2018; Staudt et al., 2014; Wijk et al., 2021) exhibiting impaired function *in vitro*. Dysregulated airway repair can also result in chronic inflammation and fibrosis (Lau et al., 2024; O'Koren et al., 2013), highlighting the need to understand and modulate basal cell behaviour for therapeutic benefit (Hynds, 2022).

Drug development is costly, with many candidates failing due to a lack of efficacy or safety concerns (Sun et al., 2022). Developing new drugs is also time-consuming; on average, it takes 10–15 years before new therapies reach patients (Berdigaliyev and Aljofan, 2020). Drug repurposing – finding new uses for approved medications – offers an efficient alternative, reducing costs and accelerating timelines by leveraging existing safety data (Ashburn and Thor, 2004; Pushpakom et al., 2019).

Airway regeneration can be modelled in primary cell cultures. Human bronchial epithelial cells (HBECs) proliferate in 2D and 3D culture systems (Hiemstra et al., 2018; Nizamoglu et al., 2023; Orr and Hynds, 2021), and differentiate in air–liquid interface (Fulcher et al., 2005) or organoid (Danahay et al., 2015) cultures. Higher-throughput adaptation of these models enables phenotypic screening (Bloom, 2021; Mayr and Bojanic, 2009). Here, we combine primary airway basal cell culture with compound screening to identify novel modulators of basal cell proliferation. Identified compounds were validated in 2D and 3D models to evaluate their effects on basal cell proliferation and differentiation.

## RESULTS AND DISCUSSION

### Compound screening in primary airway basal cells

**Fig. 1.**
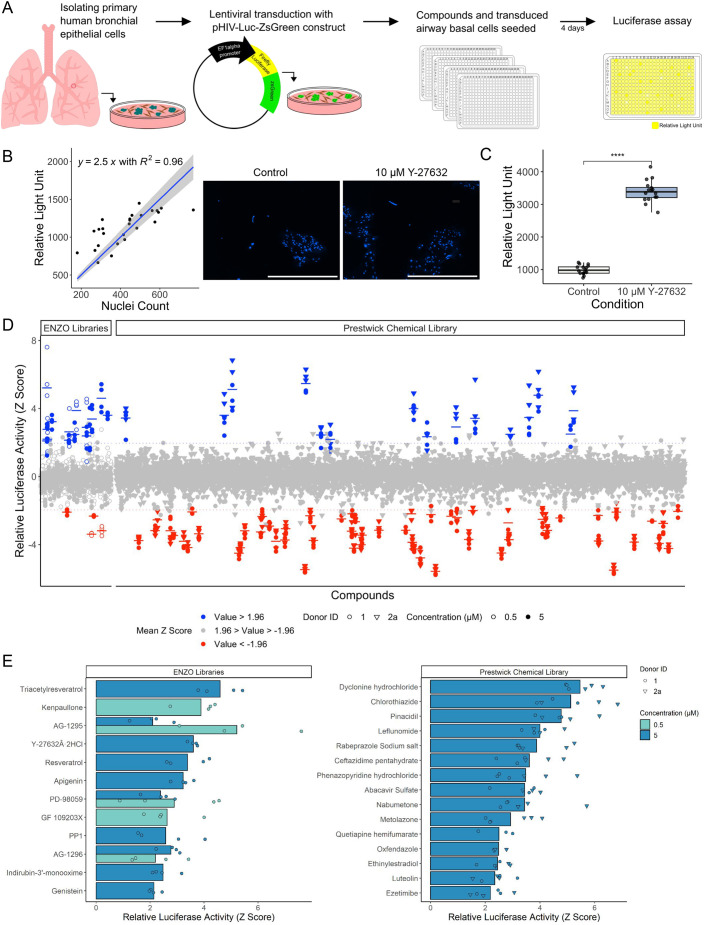
**High-throughput screening of 1429 compounds identifies compounds that modulate airway basal cell proliferation.** (A) Schematic representation of the 384-well format screening workflow. Primary human airway basal cells were transduced with a pHIV-Luc-ZsGreen lentivirus to enable luciferase monitoring of cell proliferation. Cells were seeded in 384-well format and treated with compounds from either an epigenetic modulator, protease and kinase inhibitor library (ENZO Life Sciences) or the Prestwick Chemical library for 4 days before luciferin was added to the culture medium and bioluminescence was measured. (B) Correlation between bioluminescence and nuclei count (left; *n*=26 wells, donor ID 1, representative of three cell donors tested, see Fig. S1D). Grey shading represents a 0.95 confidence interval around the linear model line. Representative images of Hoechst 33342 staining of cells following the luciferase assay (right). Scale bars: 1 mm. (C) Bioluminescence readings in the 384-well format assay comparing control or 10 μM Y-27632-treated wells (*n*=16 wells per condition, donor ID 1. ****P<0.0001 (Wilcoxon test). (D) 1429 compounds were screened in transduced airway basal cells (*n*=2 donors) in triplicate. Z scores were calculated from the relative light unit values. Hit compounds (blue) were identified as those with a mean Z score above 1.96 standard deviations of the plate average (above the top dotted line). Red colour indicates compounds with a mean Z score below 1.96 standard deviations of the plate average (below the bottom dotted line). (E) Positive hit compounds identified from screening of the ENZO (left) or the Prestwick Chemical (right) libraries.

Primary HBEC cultures were established from endobronchial biopsy specimens. Cells underwent lentiviral transduction with a pHIV-Luc-ZsGreen construct and fluorescence-activated cell sorting to purify ZsGreen-positive cells (Fig. S1A–C). This enabled 384-well format screening for modulators of basal cell proliferation by monitoring bioluminescence following the addition of luciferin to the culture medium (Fig. 1A). In validation experiments, bioluminescence signals correlated strongly with Hoechst 33342 nuclei counts (Fig. 1B; Fig. S1D), suggesting that our screening approach reports cell number. As a positive control, we tested Y-27632, a small-molecule inhibitor of Rho-associated protein kinase 1 and 2 (ROCK1 and ROCK2) (Davies et al., 2000) that is known to induce basal cell proliferation (Horani et al., 2013; Reynolds et al., 2016; Witkowski et al., 2023). As expected, the bioluminescence signal increased following the addition of Y-27632 (Fig. 1C). Calculation of the signal window (10.09) and Z′ factor (0.7) between control and Y-27632-treated wells indicated a robust assay window (Iversen et al., 2012). The screen hit threshold was set at 1.96 standard deviations above the plate mean (i.e. a mean Z score above 1.96, identifying the top 2.5% of compounds).

We screened a total of 1429 compounds from the ENZO chemical library (of epigenetic modulator and proteinase and kinase inhibitors) and the Prestwick Chemical library [of compounds that are approved for clinical use by the United States Food and Drug Administration (FDA), European Medicines Agency (EMA) and/or other agencies, and have been selected to represent broad pharmacological diversity]. We identified 27 compounds (Table S1) that increased luciferase activity above the threshold value (Fig. 1D,E), including Y-27632. An additional 61 compounds significantly reduced the number of basal cells (Fig. 1D), likely including compounds that inhibit essential processes, inhibit the cell cycle or are toxic to basal cells. Z scores for all compounds tested can be found in Table S2. The establishment of a robust primary basal cell proliferation screening protocol further demonstrates the ability to perform screening experiments in airway epithelial cells. Our approach adds to previous 96-well format screens for modulators of ciliogenesis in mouse organoids from transgenic mice (Tadokoro et al., 2014), readthrough agents in primary ciliary dyskinesia air–liquid interface cultures (Lee et al., 2021) and antiviral compounds in healthy air–liquid interface cultures (Sen et al., 2025), as well as a 384-well plate screen for inhibitors of thymic stromal lymphopoietin production performed in commercially available airway basal cells (Orellana et al., 2018).

**Fig. 2.**
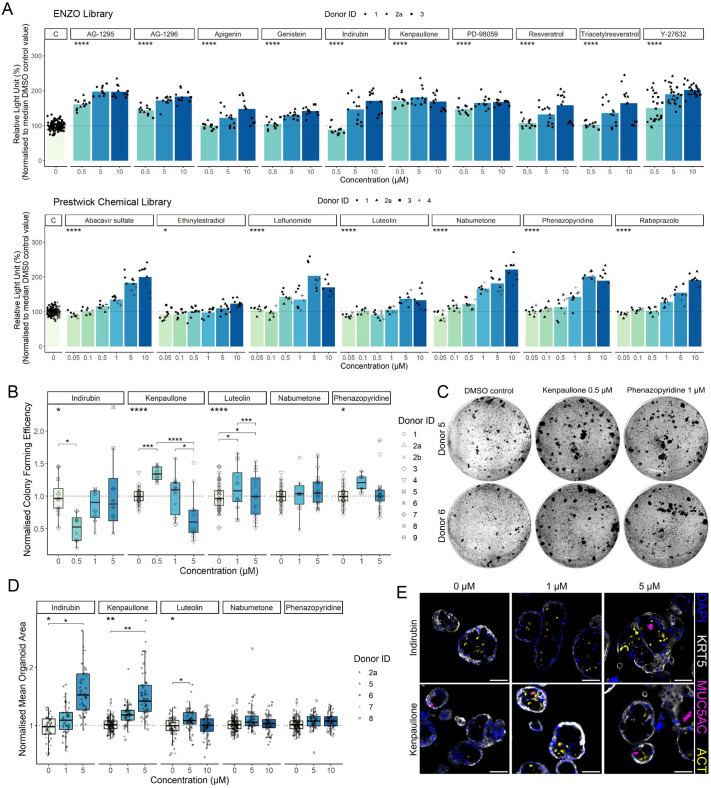
**Validation of pro-proliferative compounds in 2D and 3D primary cell culture models.** (A) Four-day concentration–response proliferation assays in primary human airway basal cells transduced with the pHIV-Luc-ZsGreen construct for compounds identified within the ENZO chemical library (upper panel) or the Prestwick Chemical library (lower panel; *n*=3 donors per compound). *P<0.05; ****P<0.0001 (two-way ANOVA was performed per compound on a linear regression model with donor and concentration as variables). (B) Airway basal cell colony formation efficiency for five validated hit compounds identified as Wnt pathway activating molecules (*n*=3–9 donors per compound). *P<0.05, ***P<0.001, ****P<0.0001 (two-way ANOVA was performed per compound on a linear regression model with donor and concentration as variables; significant Tukey's HSD values are shown in indicated pairwise comparisons). (C) Representative images from 0.5 µM kenpaullone, 1 µM phenazopyridine or vehicle-treated wells in six-well plate colony formation assays. (D) Mean organoid size following compound treatment. Data normalised to mean untreated organoid size for each donor (*n*=4–5 donors per compound). 12 replicate wells per condition per donor. *P<0.05, **P<0.01 (Friedman test was performed on the mean organoid size across all wells per donor per compound and indicated significant Nemenyi's all-pairs comparisons). (E) Representative immunofluorescence staining of organoids cultured in the presence of indirubin-3′-monoxime or kenpaullone (*n*=5 donors, donor ID 8 shown). Staining for keratin 5 (KRT5; basal cells, white), MUC5AC (mucosecretory cells, magenta) and ACT (multiciliated cells, yellow). For B and D, the box represents the 25–75th percentiles, and the median is indicated. The whiskers extend to the smallest and largest values within 1.5 times the interquartile range (IQR) from the 25th and 75th percentiles. Scale bars: 50 μm.

To validate that hit compounds accelerate basal cell proliferation, we re-purchased the test compounds from independent suppliers and performed concentration–response proliferation assays. In primary HBECs that had undergone lentiviral transduction with pHIV-Luc-ZsGreen (*n*=3 donors), 17 hit compounds (10/10 tested from the ENZO chemical library and 7/15 tested from the Prestwick Chemical library) increased luciferase activity, including Y-27632 (Fig. 2A; Fig. S2A). We also tested the effects of the ENZO library hit compounds on untransduced primary HBECs using the CellTiter-Glo assay, finding that 9/9 compounds tested induced basal cell proliferation (Fig. S2B).

We performed further validation on antiretroviral compounds within the screen. We confirmed that the nucleoside reverse transcriptase inhibitor (NRTI) abacavir sulfate, a guanosine analogue, promoted basal cell proliferation (Fig. 2A; Fig. S2C). Other NRTIs – emtricitabine (a cytosine analogue), lamivudine (a cytosine analogue) and zidovudine (a thymidine analogue) – did not have this effect (Fig. S2C; Table S2), and neither did the adenosine monophosphate (AMP) analogues tenofovir alafenamide fumarate and tenofovir disoproxil fumarate, which were not initially screened and decreased proliferation (Fig. S2C). Assessing proliferation in 3D culture conditions, three of six Prestwick Chemical library hit compounds (abacavir sulfate, leflunomide and luteolin) increased organoid (or ‘bronchosphere’) size (Fig. S2D).

Overall, this screen provides multiple avenues for future research, including compounds that could be re-purposed to promote lung regeneration.

### Wnt pathway-activating molecules promote airway basal cell proliferation

Wnt signalling is involved in the specification of lung progenitor cells (Goss et al., 2009) and the proximal–distal patterning of airways during development (Hashimoto et al., 2012; Shu et al., 2005). It also influences post-natal airway epithelial cell proliferation (Aros et al., 2020a; Hsu et al., 2014; Ievlev et al., 2022; Lynch et al., 2016), differentiation (Cooney et al., 2023; Haas et al., 2019; Malleske et al., 2018; Schmid et al., 2017) and the formation of ageing-associated glandular-like epithelial invaginations (Aros et al., 2020b). Five validated hit compounds – indirubin-3′-monoxime, kenpaullone, nabumetone, phenazopyridine and luteolin – have been previously associated with canonical Wnt signalling. Indirubins and kenpaullones inhibit GSK3β through competitive binding with ATP at the catalytic site (Leclerc et al., 2001; Zaharevitz et al., 1999). Nabumetone (a non-steroidal anti-inflammatory drug) and phenazopyridine (a urinary tract analgesic) were identified as Wnt pathway activators in a luciferase reporter cell-based screen (Costa et al., 2021), although the mechanism of pathway activation is less clear for these molecules. The role of luteolin in Wnt signalling is context dependent, upregulating β-catenin in periodontal ligament cells (Quan et al., 2019), but downregulating pathway activation in prostate cancer cell lines (Han et al., 2018).

Given the frequency of Wnt pathway-activating compounds among our hits, and the relevance of Wnt signalling to lung regeneration, we further investigated the effects of these compounds in colony formation assays finding that 0.5 μM kenpaullone, 1 μM luteolin and 1 μM phenazopyridine increased colony forming efficiency (Fig. 2B,C). In 3D organoids, the addition of indirubin-3′-monoxime, kenpaullone and luteolin significantly increased organoid size (Fig. 2D). At concentrations of these compounds that increased organoid size, the differentiation potential of basal cells was preserved, with both multiciliated cells (staining for acetylated tubulin, ACT) and mucosecretory cells (staining for mucin-5AC, MUC5AC) observed by immunofluorescence staining (Fig. 2E).

A previous screening study has identified potential lung pro-regenerative therapies by examining Prestwick Chemical library compounds in a T cell factor/lymphoid enhancer factor (TCF/LEF) family reporter assay in 3T3 mouse embryonic fibroblasts (Costa et al., 2021). Two of the five candidate drugs identified in that study, phenazopyridine and nabumetone, increased basal cell proliferation in both our screening (Fig. 1E) and validation experiments (Fig. 2A). Consistent with our human cell data, the previous study found that phenazopyridine increased mouse lung organoid frequency (Costa et al., 2021).

**Fig. 3.**
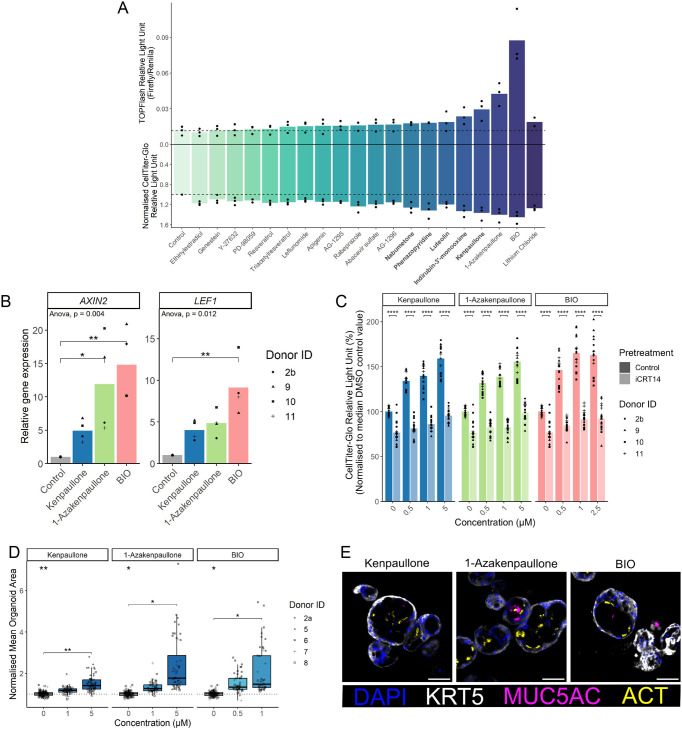
**Investigation of Wnt pathway activating compounds as promoters of airway basal cell proliferation.** (A) Wnt pathway activity in the TOPFlash assay (positive *y*-axis; compounds are ordered by Wnt pathway activity) and cell proliferation as measured by CellTiter-Glo assay (negative *y*-axis) in HBEC3-KT cells. For TOPFlash assays, cells were incubated with 10 μM of the indicated compounds (except for BIO at 1 μM and lithium chloride at 20 mM) for 24 h. For CellTiter-Glo assays, cells were plated in 384-well format and treated with 0.5 μM of the indicated compounds (except for BIO at 0.25 μM and lithium chloride at 10 mM) for 3 days. Points are the mean value of 5–8 replicate wells per experiment (*n*=3 experiments). Dashed lines show mean control values. Data are normalised to control wells containing the highest concentration of DMSO used in the experimental conditions. (B) qPCR analysis of the Wnt target gene *AXIN2* or *LEF1*. Primary human airway basal cells (*n*=4 donors) were treated with the indicated compounds for 24 h. Relative expression normalised to the control condition is shown. *P<0.05, **P<0.01 (one-way ANOVA test was performed per target gene and significant Tukey's HSD values are shown). (C) Effect of the Wnt pathway inhibitor iCRT14 on primary basal cell proliferation in the presence of kenpaullone, 1-azakenpaullone or BIO. Cells were pre-treated with 10 μM iCRT14 or DMSO control for 24 h before the addition of the Wnt pathway activating compounds. Relative growth was assessed after 6 days using the CellTiter-Glo assay (*n*=4 donors). ****P<0.0001 (Wilcoxon tests were performed with Holm correction for multiple testing per compound). (D) Mean organoid size per well with 12 replicate wells per condition normalised to mean control organoid size for each donor (*n*=5 donors). *P<0.05, **P<0.01 (Friedman test was performed on the mean organoid size across all wells per donor per compound and significant Nemenyi's all-pairs comparisons are shown). Control well data are repeated per compound facet and kenpaullone data is repeated from Fig. 2C. (E) Immunofluorescence staining showing KRT5 (basal cells, white), MUC5AC (mucosecretory cells, magenta), and ACT (multiciliated cells, yellow) in organoids following culture in medium containing the indicated compounds (*n*=5 donors, donor ID 8 shown). For D, the box represents the 25–75th percentiles, and the median is indicated. The whiskers extend to the smallest and largest values within 1.5 times the interquartile range (IQR) from the 25th and 75th percentiles. Scale bars: 50 μm.

To assess Wnt pathway activation by hit compounds, we transfected the immortalised basal cell line HBEC3-KT (Ramirez et al., 2004) with TOPFlash plasmids, in which firefly luciferase expression is controlled by a minimal promoter sequence and multiple TCF-binding sites. Given that more potent and selective derivatives were available for two of the Wnt-activating screening hits, kenpaullone and indirubin-3′-monoxime, the hit compounds were expanded to include 1-azakenpaullone (Kunick et al., 2004) and 6-bromoindirubin-3-oxime (BIO) (Meijer et al., 2003), respectively. Five putative Wnt modulatory screen compounds, along with 1-azakenpaullone and BIO, activated Wnt signalling in airway basal cells to a similar or greater extent than lithium chloride (Fig. 3A), which acted as a positive control (Klein and Melton, 1996). Wnt-activating compounds induced higher expression levels of the Wnt target genes *AXIN2* and *LEF1* (Fig. 3B) but not *CCND1* or *MYC* (Fig. S3A) in primary HBECs. Pretreatment with iCRT14, a small-molecule inhibitor that blocks the transcriptional activity of β-catenin (Catrow et al., 2015), prevented the increase in proliferation observed in primary HBECs in response to kenpaullone, 1-azakenpaullone and BIO (Fig. 3C), suggesting that the mechanism of action is canonical Wnt pathway activation. Finally, these compounds increased organoid size in a concentration-dependent manner (Fig. 3D), and organoids cultured in medium containing any of the three compounds contained ACT+ multiciliated cells and MUC5AC+ mucosecretory cells (Fig. 3E), indicating that they retained airway differentiation potential.

### 1-Azakenpaullone activates Wnt signalling in mouse trachea and lung *in vivo*

**Fig. 4.**
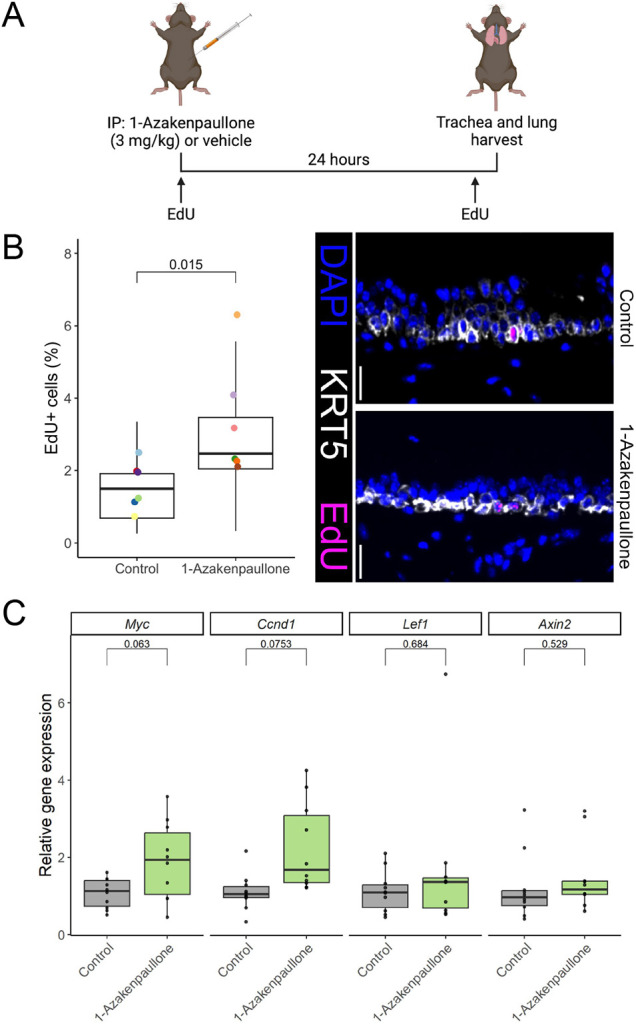
**1-Azakenpaullone induced tracheal proliferation and the expression of Wnt target genes *in vivo*.** (A) Schematic representation of the *in vivo* experimental design. Male C57Bl/6 mice were administered EdU, and 1-azakenpaullone (3 mg/kg of body weight) or vehicle control by intraperitoneal (IP) injection. After 24 h, mice were euthanised and tracheas and lungs were harvested for analysis. Created in BioRender by Hynds, R.E., 2025. https://BioRender.com/z01x697. This figure was sublicensed under CC-BY 4.0 terms. (B) Tracheal proliferation as assessed by EdU staining in mice treated with 1-azakenpaullone or vehicle control (*n*=6 mice per condition). Quantification (left) was performed from immunofluorescence staining (right). KRT5 (basal cells, white) and EdU (magenta). Scale bars: 20 μm. Points indicate mean values per mouse and boxplot represents data from all images. Wilcoxon test *P*-value shown. (C) Expression of the Wnt target genes *Myc*, *Ccnd1*, *Lef1* and *Axin2* in mouse trachea as determined by qPCR. Relative expression was normalised to control animals*.* Wilcoxon tests were performed per target gene (*n*=10 mice per condition) and *P*-values are shown. For B and C, the box represents the 25–75th percentiles, and the median is indicated. The whiskers extend to the smallest and largest values within 1.5 times the interquartile range (IQR) from the 25th and 75th percentiles.

Kenpaullone, indirubin-3′-monoxime and their derivatives 1-azakenpaullone and BIO consistently promoted basal cell proliferation in our *in vitro* studies with the latter two being most effective. Although BIO is a potent GSK3β inhibitor, it also targets cyclin-dependent kinases (CDKs) (Meijer et al., 2003). Given that 1-azakenpaullone has a greater selectivity for GSK3β than BIO (Kunick et al., 2004) and it demonstrated a stronger enhancement of cell proliferation using a 3D organoid model than kenpaullone (Fig. 3D), we assessed the potential of 1-azakenpaullone to modulate airway basal cell proliferation and Wnt pathway activation *in vivo*. We administered 1-azakenpaullone (3 mg/kg of body weight) or PBS along with EdU intraperitoneally in C57Bl/6 mice (Fig. 4A). At 24 h following administration, we found an increased proportion of EdU+ tracheal epithelial cells in mice treated with 1-azakenpaullone, indicating increased cell proliferation (Fig. 4B). We also found trends towards increased expression of the Wnt target genes *Myc* and *Ccnd1*, but not *Lef1* or *Axin2*, in tracheal cells following 1-azakenpaullone administration (Fig. 4C). In lung tissue from the same mice, we found a significant increase in *Myc* but not *Ccnd1*, *Lef1* or *Axin2* expression (Fig. S3B), which was accompanied by increased protein expression of Myc and cyclin D1 (Fig. S3C).

Overall, our study confirms canonical Wnt pathway activation as a pro-proliferative signal for airway epithelial cells. 1-Azakenpaullone administration to uninjured mice activated the Wnt pathway and increased tracheal epithelial cell proliferation. Wnt signalling during airway regeneration is complex and spatiotemporally regulated, with Wnt sources differing between the homeostatic (epithelial-derived) and injured (stromal-derived) states (Aros et al., 2020b). Non-canonical Wnt signalling can counteract the canonical pathway to inhibit alveolar regeneration (Baarsma et al., 2017). Although canonical Wnt signalling maintains ΔNp63-expressing stem cells, dysregulation can lead to basal cell hyperplasia (Aros et al., 2020a) or squamous differentiation (Zhang et al., 2023), and human airway premalignant and malignant lesions show evidence of Wnt pathway activation (Aros et al., 2020a). Thus, future efforts to manipulate Wnt signalling for airway regeneration must consider differences in its effects during injury versus homeostasis, and the desired magnitude and duration of pathway activation.

## MATERIALS AND METHODS

### Cell culture

Human tissue was obtained from regions of histologically normal airway via endobronchial biopsy or as segments of normal lung following lobectomy procedures with informed consent. Ethical approval was obtained through the National Research Ethics Committee (REC reference 18/SC/0514). Research on human cells was conducted in accordance with the principles expressed in the Declaration of Helsinki. Cell culture plates and flasks were Nunc branded and purchased from Thermo Fisher Scientific. Cells were cultured in 37°C incubators with 5% CO_2_. Cells were regularly tested to ensure the absence of mycoplasma contamination.

3T3-J2 mouse embryonic fibroblasts (a gift from Fiona Watt, King's College London, UK) were cultured in Dulbecco's modified Eagle's medium (DMEM; Gibco, 41966) with 9% bovine serum (26170043, Gibco) and 1× penicillin-streptomycin. Feeder layers were generated between passages 9–12. Cells were mitotically inactivated by treatment with 4 μg/ml mitomycin C (M4287, Sigma-Aldrich) in cell culture medium for 3 h. Cells were trypsinised and plated at 20,000–30,000 cells/cm^2^. Epithelial cells were added the following day.

Endobronchial biopsies and lobectomy tissue (Table S4) arrived in the laboratory on ice in transport medium [αMEM (22561, Gibco) supplemented with 1× penicillin-streptomycin (15140-122, Gibco), 10 μg/ml gentamicin (15710-049, Gibco) and 250 ng/ml amphotericin B (BP264550, Fisher Bioreagents)].

To generate single-cell suspensions, biopsies and airway tissue were incubated in 16 U dispase (354235, Corning) in RPMI (21875, Gibco) for 20 min at room temperature and then mechanically disrupted using sterile forceps. This was followed by incubation in 0.1% trypsin/EDTA (59418C, Sigma-Aldrich) for 30 min at 37°C. Both dispase and trypsin/EDTA incubations were neutralised with FBS. The digested tissues were filtered through a 100 μm cell strainer (352360, Falcon). Single cell suspensions were centrifuged at 300× ***g*** for five minutes and resuspended in relevant media for counting and plating.

Epithelial cell culture medium (Butler et al., 2016; Hynds et al., 2019; Liu et al., 2012) consisted of a 3:1 ratio of DMEM supplemented with 9% fetal bovine serum (FBS) (10270106, Gibco) and 1× penicillin-streptomycin with Ham's F12 (21765, Gibco) supplemented with 5 μM Y-27632 (T1725, Tebu-Bio), 25 ng/ml hydrocortisone (H0888, Sigma-Aldrich), 0.125 ng/ml epidermal growth factor (EGF; PHG0315, Gibco), 5 μg/ml insulin (I5500, Sigma-Aldrich), 0.1 nM cholera toxin (C8052, Sigma-Aldrich), 250 ng/ml amphotericin B (BP2645, Fisher Bioreagents) and 10 μg/ml gentamicin.

FAD medium consisted of DMEM/Ham's F12 in a 3:1 ratio supplemented with 10% FBS, 1× penicillin-streptomycin, 180 μM adenine (A3159, Sigma-Aldrich), 0.5 μg/ml hydrocortisone, 10 ng/ml EGF, 5 μg/ml insulin, 0.1 nM cholera toxin, 2 nM T3 (T5516, Sigma-Aldrich), 10 μg/ml gentamicin and 250 ng/ml amphotericin B.

The differential trypsin sensitivity of 3T3-J2 fibroblasts and epithelial cells was used to isolate epithelial cell populations from co-cultures. Co-cultures were washed with phosphate-buffered saline (PBS; D8537, Sigma-Aldrich) and 0.05% trypsin-EDTA (25300054, Gibco) was added for 2 min to remove the 3T3-J2 fibroblasts, which are more sensitive to trypsin than the strongly adherent epithelial cells. Flasks were then washed with PBS and 0.05% trypsin-EDTA was added a second time for 5–10 min to detach the epithelial cells.

The HBEC3-KT cell line (CRL-4051, ATCC) was cultured in Airway Epithelial Cell Basal Medium (PCS-300-030, ATCC) supplemented with the Bronchial Epithelial Cell Growth Kit (PCS-300-040, ATCC) or in Keratinocyte SFM (17005-042, Thermo Fisher Scientific), a serum-free medium supplemented with recombinant EGF and bovine pituitary extract. Cells were passaged by trypsinisation with 0.05% trypsin-EDTA. Cells were centrifuged at 300 ***g*** for 5 min to ensure trypsin removal.

Cells cultured in epithelial cell culture medium were cryopreserved in 50% cell culture medium, 42.5% ProFreeze (BP12-769E, Lonza) and 7.5% DMSO (D2650, Sigma-Aldrich). Cells cultured in FAD medium were frozen in 1x Cell Freezing Medium-Glycerol (C6039, Merck). Cryovials were transferred to a CoolCell and placed in an −80°C freezer to cool overnight. Cryopreserved cells were then stored at −150°C.

### Virus production

HEK293Ts were cultured in DMEM supplemented with 10% FBS and 1× penicillin-streptomycin. Antibiotics were omitted for one passage prior to and during transfection.

Viral supernatants were produced by co-transfecting HEK293T cells at 70-80% confluency in a T175 flask with 20 μg pHIV-Luc-ZsGreen (deposited by Bryan Welm; Addgene plasmid #39196), 13 μg pCMVR8.74 (deposited by Didier Trono; Addgene plasmid #22036) and 7 μg pMD2.G (deposited by Didier Trono; Addgene plasmid #12259) using JetPEI (101000053, Polyplus Transfection) and following the manufacturer's protocol. Viral supernatants were collected 48 h and 72 h post-transfection and filtered through a 0.45 μm filter (6780-2504, Whatman). Supernatant was combined with PEGit concentrator (5×) (LV810A-1, System Biosciences) overnight at 4°C and centrifuged at 1500 ***g*** for 45 min at 4°C. The supernatant was removed, and the viral pellet was resuspended in 1/10th of the original supernatant volume in DMEM supplemented with 25 mM HEPES. Concentrated supernatants were stored at −80°C until use.

### Lentiviral transduction of primary airway basal cells

Epithelial cells were plated at 150,000 cells per well in a six-well plate on 3T3-J2 feeder layers. The following day, cells were transduced with the addition of concentrated virus (Fig. S1C) and polybrene (4 μg/ml; Santa Cruz Biotechnology) to the cell culture medium. Medium was replaced 7 h after the addition of the virus. After one passage, transduced cells were enriched by fluorescence-activated cell sorting (FACS) using their expression of ZsGreen. Single-cell suspensions of epithelial cells were collected by differential trypsinisation and were filtered through a 70 μm strainer (352350, Falcon), centrifuged 300 ***g*** for 5 min, and resuspended in FACS buffer (PBS, 1% FBS, 25 mM HEPES and 1 mM EDTA). FACS experiments were performed using either a BD FACS Aria or a BD FACS Aria Fusion sorter.

### Compound screening in primary airway basal cells

1429 compounds from the ENZO library (159 compounds) and the Prestwick Chemical library (1277 compounds) were tested. Seven compounds were shared between both libraries, giving a total of 1429 unique compounds in our study. Compounds were pre-plated in a 384-well format at 2 mM in DMSO. The plates were stored in a nitrogen-purged StoragePod (Roylan Developments) in 5% humidity with 5% O_2_. Potential hit compounds were re-ordered for validation experiments. Compounds for validation experiments (Table S3) were reconstituted as 10 mM stocks in DMSO, aliquoted and stored at −20°C to avoid multiple freeze–thaw cycles.

Primary airway basal cells transduced with the pHIV-Luc-ZsGreen construct were expanded in epithelial cell culture medium and cultured for one passage in FAD medium prior to plating in 384-well plates (142761, Thermo Fisher Scientific). First, compound libraries and DMSO were added to the assay plate using an ECHO 550 liquid handler (Labcyte). 2000 cells/well were then seeded in FAD medium using a microplate washer/dispenser (BioTek EL406, Agilent) under sterile conditions. After 4 days of culture, medium was removed, and luciferin was added to cells at 150 μg/ml in FAD medium using the microplate washer/dispenser. After 10 min, bioluminescence was measured using a Envision II plate reader (PerkinElmer). Relative light units were normalised to the DMSO control well values.

For nuclei visualisation, medium was removed and 2 µg/ml Hoechst 33342 (H3570, Invitrogen) in FAD medium was added. After 10 min the wells were imaged and nuclei counted using a Cytation 3 imaging reader (Biotek).

### Cell proliferation experiments

For experiments on untransduced HBECs, cells were expanded on 3T3-J2 feeder cells in FAD medium. For proliferation assays, HBECs (without feeder cells) or HBEC3-KT cells were seeded at 2000 cells/well in 384-well plates and compounds were added at the time of seeding using a E1-ClipTip multichannel pipette (Thermo Fisher Scientific). After the indicated number of days in culture, cells were lysed using CellTiter-Glo (G7570, Promega) reagents, according to the manufacturer's protocol. Bioluminescence was measured using a Envision II plate reader.

To investigate the effect of inhibiting canonical Wnt pathway activation, primary airway basal cells that had been cultured in FAD medium prior to the assay were seeded at 2000 cells/well in a 384-well plate in FAD medium containing 10 μM inhibitor of β-catenin responsive transcription 14 (iCRT14; HY-16665, MedChemExpress). The following day hit compounds were added to the wells. After 6 further days in culture, the cell viability was measured using the CellTiter-Glo protocol; the relative light unit was measured by a Envision II plate reader.

### TOPFlash assay

The HBEC3-KT cell line was seeded in 6-well plates at 900,000 cells/well. The following day cells were co-transfected with the pRL SV40 construct (E2231, Promega) using jetOPTIMUS (101000051, Polyplus) along with either the M50 Super 8x TOPFlash (deposited by Randall Moon, Addgene plasmid #12456; Veeman et al., 2003) or the M51 Super 8x FOPFlash – an inactive TOPFlash mutant (deposited by Randall Moon, Addgene plasmid #12457) according to the manufacturer's instructions. Medium was changed after 6 h. The next day, cells were seeded at 20,000 cells/well in a 384-well plate and compounds were added after 3 h. Luciferase activity was measured using Dual-Glo luciferase assay system by a Envision II plate reader 48 h after compound addition.

### Colony formation assays

To calculate colony forming efficiency, primary airway basal cells were seeded at 1000 cells/well in FAD medium on six-well plates that had been pre-coated with collagen I and 3T3-J2 feeder cells (Hynds et al., 2018). Experimental compounds or an equivalent DMSO volume for control wells were added the following day. Medium was changed twice per week. On day 10, cells were fixed in 4% PFA for 20 min and stained with crystal violet solution (HT90132, Sigma-Aldrich) for 15 min at room temperature. Plates were washed thoroughly with running water and dried overnight. Plates were scanned using an Epson Perfection V700 PHOTO Scanner. Colonies were manually counted using a brightfield microscope. Colonies were defined as contiguous groups of more than ten cells. Colony forming efficiency (%) was calculated as (number of colonies counted/number of cells seeded)×100.

### Organoid culture

Organoids (‘bronchospheres’) were cultured as per previously published methods (Hynds et al., 2019). Differentiation medium consisted of 50% DMEM and 50% Bronchial Epithelial Cell Growth Basal Medium (CC-3171, Lonza) supplemented with the Bronchial Epithelial Cell Growth Medium SingleQuots (except amphotericin B, triiodothyronine and retinoic acid; CC-4175, Lonza). Medium was supplemented with 100 nM all-trans retinoic acid (R2625, Sigma-Aldrich) at time of use. Briefly, wells of an ultra-low attachment 96-well plate (3474, Corning) were coated with 30 μl 25% Matrigel (354230, Corning) in bronchosphere medium and returned to the incubator at 37°C for 30 min. Epithelial cells were seeded at 2500 cells/well in 65 μl 5% Matrigel in bronchosphere medium containing 5 μM Y-27632. Organoids were fed by the addition of 50 μl bronchosphere medium supplemented with experimental compounds at the indicated concentrations or equivalent DMSO volume for control wells on days 3, 10 and 17 of culture.

On day 21, whole-well images were taken with a 10× magnification objective, and image analysis was performed with OrgaQuant (Kassis et al., 2019). Organoids were collected in ice-cold PBS and centrifuged at 300 ***g*** for 5 min. Organoids were fixed in 4% PFA on ice for 30 min and then centrifuged at 400 ***g*** for 5 min. Organoids were then washed with ice-cold PBS and transferred to a well of a V-bottomed 96-well plate. The plate was centrifuged at 400 ***g*** for 5 min and organoids were resuspended in 120 µl pre-warmed HistoGel (HG4000012, Fisher Scientific). After 10 min on ice, the gel was transferred to 70% ethanol at 4°C before being processed.

### Immunofluorescence staining

Formalin-fixed, histogel-embedded organoids and formalin-fixed tissues were processed in a Leica TP1050 tissue processor. Samples were embedded in type 6 paraffin wax (8336, Epredia) using an embedding station (Sakura Tissue-TEK TEC) and 5 µm sections were cut on a Microm HM 325 microtome. Slides were dewaxed using an automated protocol. Slides were washed in PBS and a hydrophobic ring was drawn around the sample using an ImmEdge pen (H-4000, Vector Laboratories). Sections were blocked with 1% bovine serum albumin (BSA; 1.12018.0100, Merck), 5% normal goat serum (NGS; Abcam, ab7481) and 0.1% Triton X-100 (X-100, Sigma-Aldrich) in PBS for 1 h at room temperature. Primary antibodies raised against TP63 (ab124762, Abcam; 1:300), KRT5 (905901, Biolegend; 1:500), MUC5AC (M5293, Sigma-Aldrich; 1:500) and ACT (T6793, Sigma-Aldrich; 1:500) were diluted in blocking buffer and applied to slides overnight at 4°C. Slides were washed twice in PBS. For EdU detection the Click-iT™ EdU Cell Proliferation Kit for Imaging with Alexa FluorTM 488 dye (C10337, Thermo Fisher Scientific) was used according to the manufacturer's protocol prior to secondary antibody application. Species appropriate secondary antibodies conjugated to Alexa Fluor dyes were diluted 1:1000 in 5% NGS, 0.1% Triton X-100 in PBS and applied to slides for 3 h at room temperature in the dark. 100 ng/ml DAPI (D9542, Sigma-Aldrich) in PBS was applied to the slides for 20 min. Slides were washed twice in PBS and a coverslip was applied manually with Immu-Mount (9990402, Thermo Fisher Scientific). Images were acquired using a Leica DMi8 fluorescence microscope.

### qPCR

Total RNA was extracted using the Quick-RNA Miniprep Plus kit (Zymo Research) according to the manufacturer's instructions. cDNA was synthesised using qScript cDNA Supermix (95048-100, Quantabio). Quantitative (q)PCR was performed with SYBR Green (4367659, Thermo Fisher Scientific) using QuantStudio5™ Real-time PCR system (Thermo Fisher Scientific). A list of primers used for qPCR is provided in Table S5. Relative gene expression was calculated using a comparative CT method with reference genes (*RPS13* and/or *GAPDH* for human genes, *Actb* and/or *Hprt1* for mouse genes). Statistics were performed on dCt values.

### *In vivo* experiment

Animal studies were approved by the University College London Biological Services Ethical Review Committee and licensed under UK Home Office regulations (project licence PP2060881). Male mice on the C57Bl/6 background (11-13 weeks of age) were administered one dose of 1-azakenpaullone (3 mg/kg of body weight) or vehicle control (3% DMSO, 50% PEG300, 5% Tween-80 and 42% PBS) by intraperitoneal injection. Mice were group-housed and randomly assigned to either vehicle or treatment groups, with individuals from both groups co-housed in the same cages to control for environmental effects. After 24 h, mice were euthanised by overdose of pentobarbital for isolation of tracheal and lung tissue. To determine cell proliferation, EdU (Merck) was administered with 1-azakenpaullone 24 h before harvesting (50 µg/g of body weight EdU), and alone 2 h before harvesting (50 µg/g of body weight EdU).

For qPCR analysis of tracheal epithelium, dissected tracheas were first incubated in dispase (50 Unit/ml, 354235, Corning) for 40 min at 37°C. Following incubation, the epithelial layer was flushed from the trachea with 10 ml ice-cold PBS into a 15 ml tube using a needle and syringe. Cells were centrifuged at 400 ***g*** and resuspended in RNA lysis buffer for RNA extraction. Lung tissue was lysed directly in RNA lysis buffer from the Quick-RNA Miniprep Plus kit (Zymo Research) using a microtissue homogeniser.

To enable immunofluorescence staining of the tracheal epithelium, dissected tracheas were fixed in 4% PFA at 4°C overnight and then stored in 70% ethanol at 4°C before being processed.

To automate quantification analyses, a macro was created in Fiji software (Schindelin et al., 2012) to mask and quantify the count and area of immunofluorescent staining. The macro was run by two researchers independently to account for thresholding differences between users. The researchers were unaware of the experimental conditions during quantification. The mean EdU proportion per section was calculated.

### Western blotting

Mouse lungs were snap frozen on dry ice at the time of collection. Lungs were thawed on ice, minced with a scalpel, homogenised and lysed in RIPA lysis buffer with protease inhibitors (cOmplete Ultra tablets, 5892970001, Merck) to extract protein. 30 μg of protein samples were separated by SDS-PAGE and transferred onto nitrocellulose membranes using an iBlot2 Dry Blotting System (Thermo Fisher Scientific). Membranes were incubated with anti-cyclin-D1 (1:1000, 55506, Cell Signaling Technologies), anti-Myc (1:1000, ab32072, Abcam), or anti-α-tubulin (1:2000, 9099, Cell Signaling Technologies) antibodies, washed and incubated with species-appropriate horseradish peroxidase (HRP)-conjugated secondary antibodies. After incubating with the substrate (Immobilon Crescendo Western HRP Substrate; WBLUR0500, Millipore), chemiluminescent signals were visualised using an iBright FL1500 imaging system (Thermo Fisher Scientific). Quantification of bands was performed using Fiji. Uncropped images of western blots are shown in Fig. S4.

### Data visualisation

Fig. 1A was produced using Inkscape. Fig. 4A was created in BioRender (https://BioRender.com/z01x697). Analyses and data visualisation were performed in RStudio (v2024.09.0.375) with the tidyverse [v2.0.0] packages: dplyr (dplyr.tidyverse.org/, v1.1.4), tidyr (tidyr.tidyverse.org/, v1.3.1), tibble (tibble.tidyverse.org/, v3.2.1), ggplot2 (ggplot2.tidyverse.org/, v3.5.1), ggpubr (rpkgs.datanovia.com/ggpubr/, v.0.6.0), rstatix (https://rpkgs.datanovia.com/rstatix/, v0.7.2) and the colour maps Viridis (github.com/sjmgarnier/viridis/, v0.6.5) or RColorBrewer (v1.1.3). All statistical tests are two-tailed.

## Supplementary Material



10.1242/joces.263487_sup1 Supplementary information

Table S2. Z Scores for all screened compounds.
