# Supplementary material for: Compound screening in human airway basal cells identifies Wnt pathway activators as potential pro-regenerative therapies
Source: J Cell Sci. 2025 Apr 14;138(7):jcs263487. doi: 10.1242/jcs.263487 (PMC12045047; doi:10.1242/jcs.263487)
Supplement: Supplementary information [file joces-138-263487-s1.pdf]

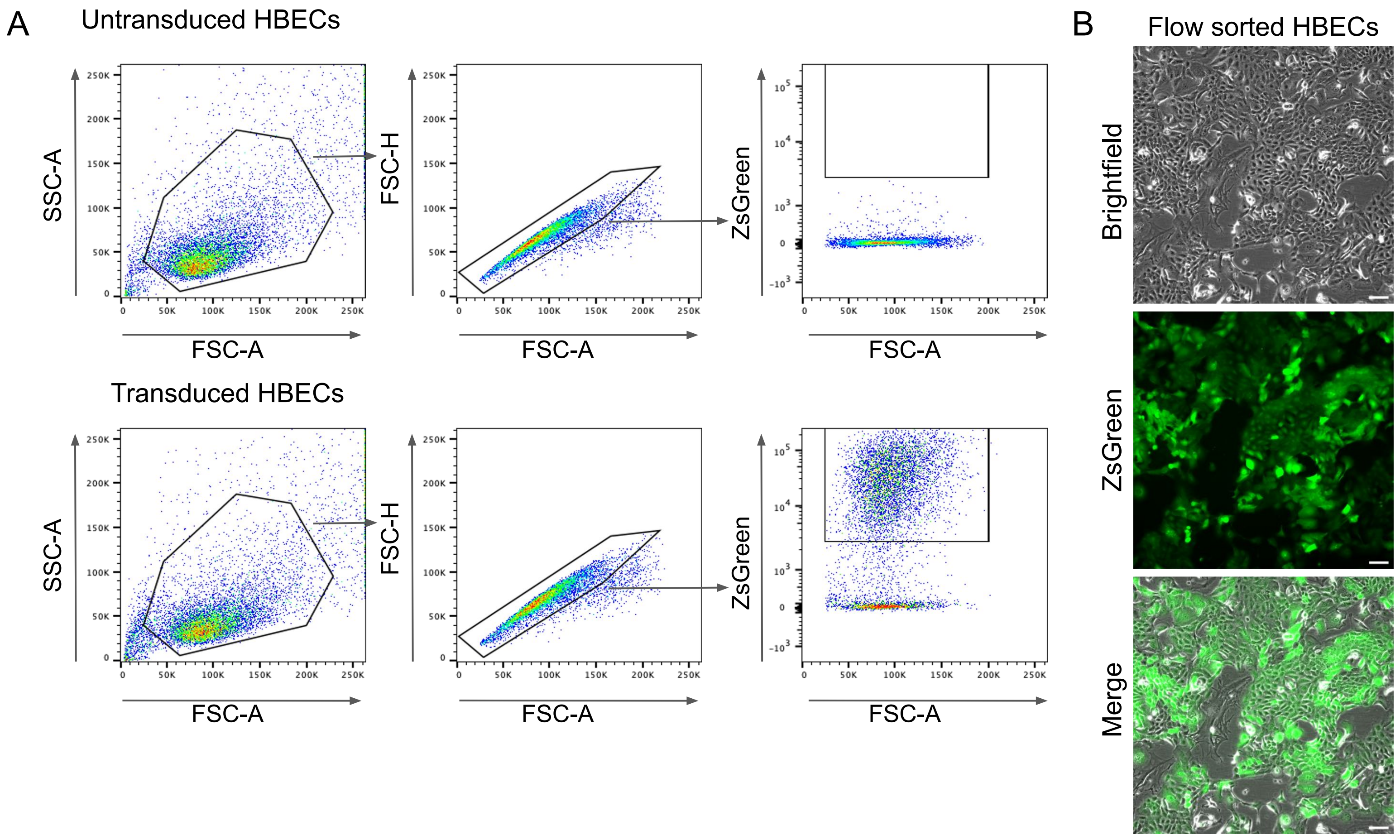

C

Donor ID	MOI	Transduction efficiency (%) (ZsGreen+ of single cell population)	Number of cells sorted
1	2	31.0	270,000
2a	6	65.5	200,000
3	2	68.1	100,000
4	6	72.1	75,000

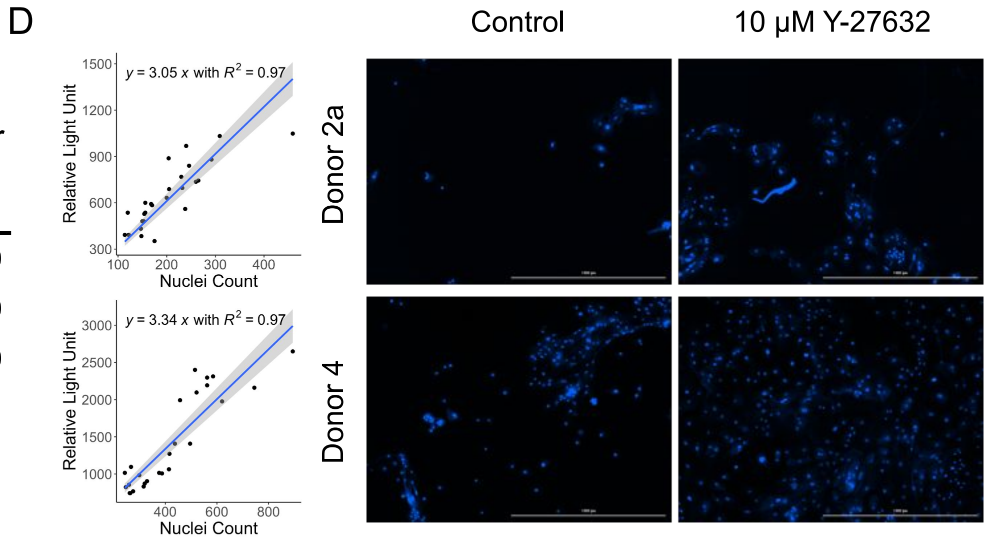

Fig. S1. Transduction and validation of zsGreen-Luciferase transduced primary airway basal cells for compound screening. A) Gating strategy for flow sorting of ZsGreen+ population with data shown from donor 4. **B)** Representative brightfield and fluorescence images of flow sorted human bronchial epithelial cells (HBECs) from a single donor. Scale bars = 50 µm. **C)** Transduction efficiency for each HBEC donor following lentiviral transduction with the pHIV-Luc-Zsgreen construct. **D)** Correlation between bioluminescence and nuclei count (left). Each data point is a reading from one well (n = 26 wells per donor). Representative images of Hoechst 33342 staining of cells following the luciferase assay (right). Scale bars = 1 mm. Data relate to Figure 1B, showing two additional primary cell donors.

Journal of Cell Science • Supplementary information

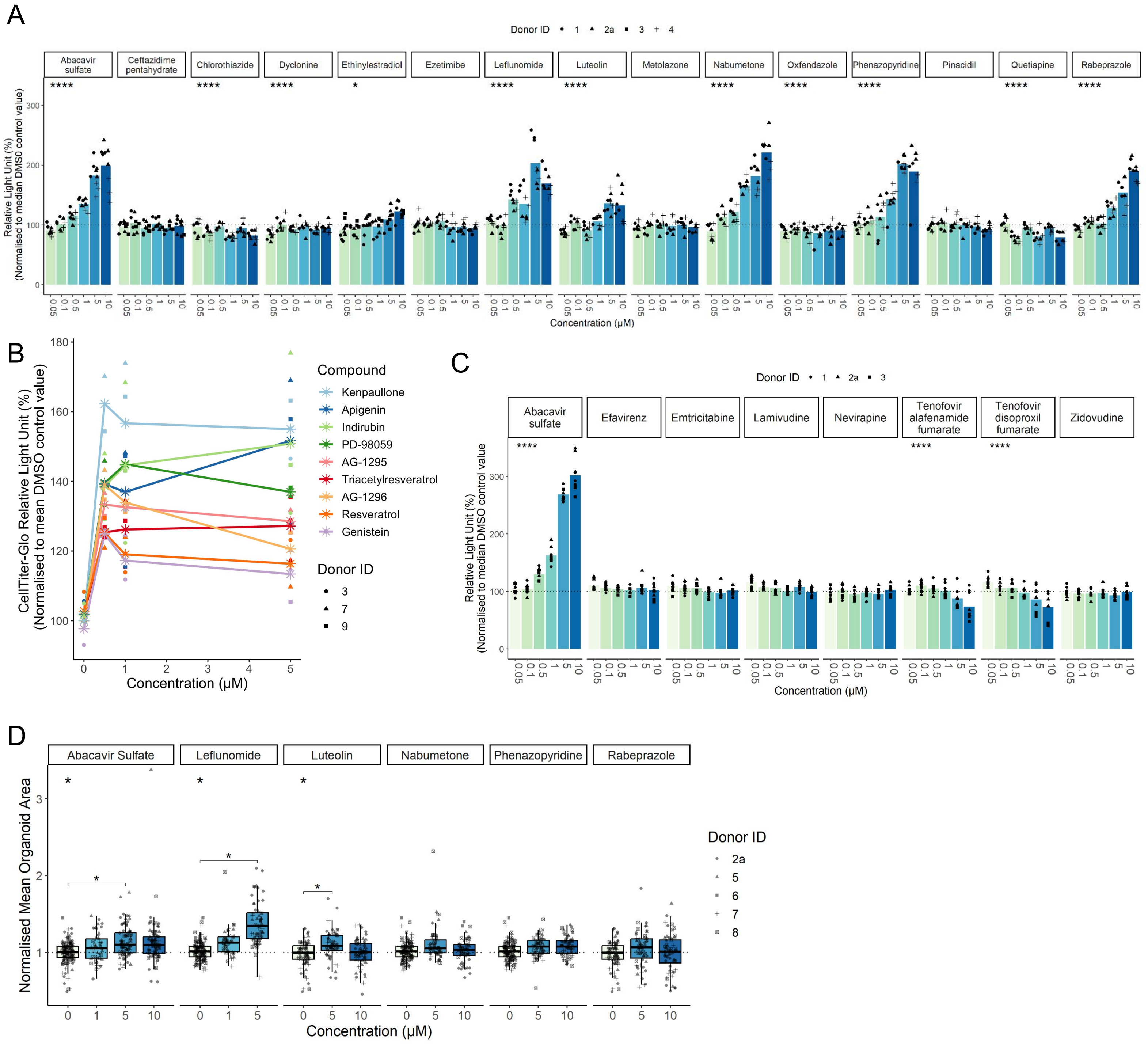

Fig. S2. Related to Figure 2. A) Four-day concentration-response proliferation assays in primary human airway basal cells transduced with the pHIV-Luc-zsGreen construct for compounds identified within the Prestwick Chemical library (n = 3 donors per compound). An ANOVA was performed per compound. Compounds with a significant difference are repeated from Fig. 2A. **B)** A concentration-response experiment on hit compounds from the ENZO chemical library. Untransduced primary human bronchial epithelial cells (n = 3 donors) were cultured with the indicated concentrations of screen hit compounds for six days. Relative cell growth was assessed using the CellTiter-Glo assay. * denotes mean value at each concentration tested. **C)** Four-day concentration-response proliferation assays in primary human airway basal cells transduced with the pHIV-Luc-ZsGreen construct for antiretroviral compounds (n = 3 donors per compound). An ANOVA was performed per compound. **D)** Quantification of mean organoid size per well with 12 replicate wells per condition were normalised to mean control well organoid size for each donor (n = 5 donors). A Friedman test was performed on the mean organoid size across all wells per donor per compound and significant Nemenyi's all-pairs comparisons are shown. Control well data are repeated per compound facet, data for Luteolin, Nabumetone and Phenazopyridine are repeated in 2C. * = p<0.05, **** = p<0.0001.

Journal of Cell Science • Supplementary information

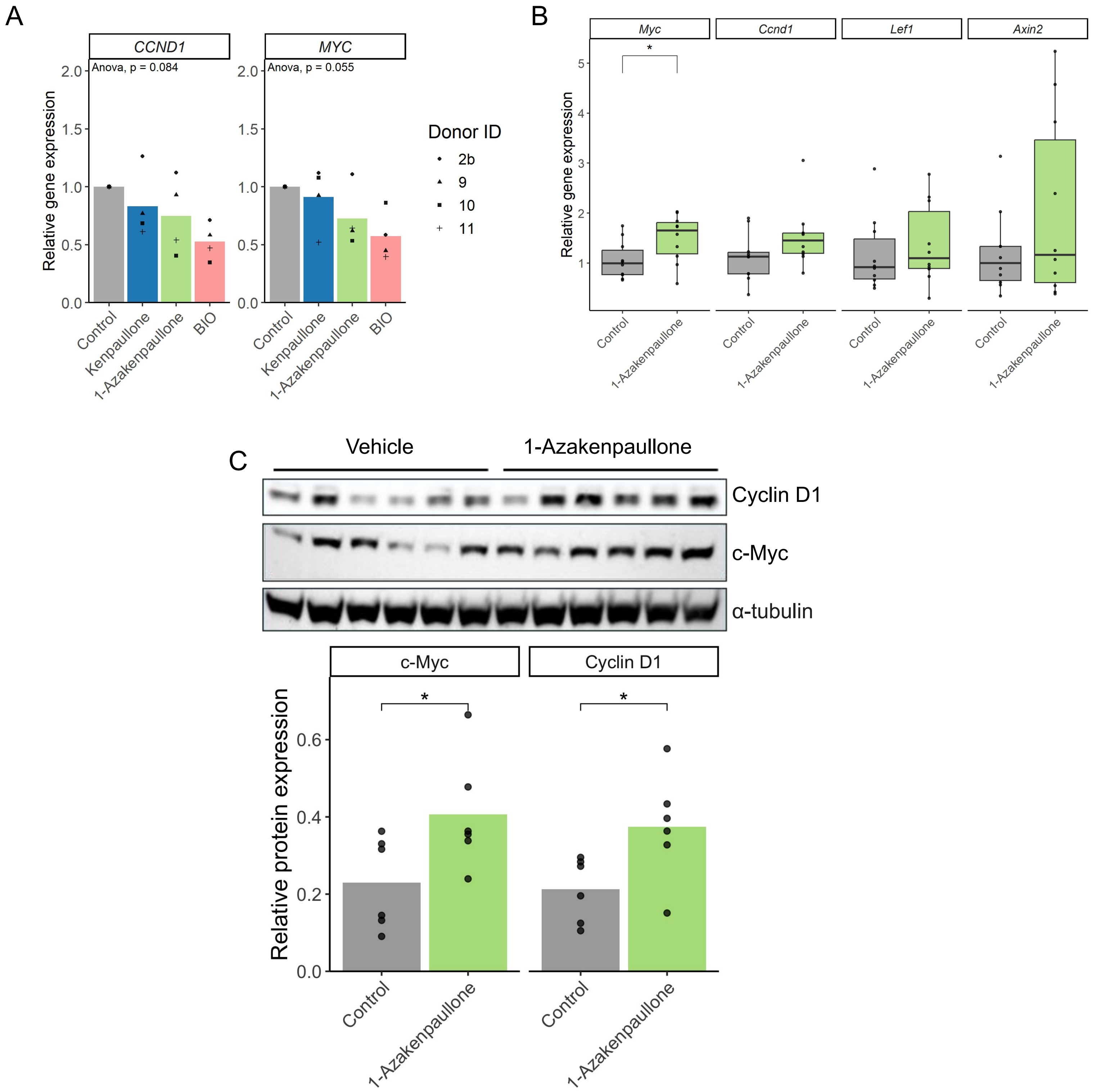

Fig. S3. Additional data relating to Figure 3 and Fig. 4.

A) qPCR analysis of the Wnt target genes *CCND1 and MYC* in primary human airway basal cells (n = 4 donors) treated with compounds for 24 hours. Relative expression normalised to the control condition is shown. An ANOVA test was performed per target gene. **B)** Expression of the Wnt target genes *Myc, and Ccnd1, Lef1 and Axin2* in mouse lungs following treatment with vehicle control or 1-azakenpaullone as determined by qPCR. Relative expression normalised to the control condition is shown. A Wilcoxon test was performed (n = 10 mice per condition). **C)** Expression of Wnt target proteins in lung tissue following treatment with vehicle control or 1-azakenpaullone as determined by Western blot (n = 6 mice per condition). Alpha-tubulin is shown as a loading control. The bands were quantified using Fiji and the relative expression of c-Myc and CyclinD1 normalised to alpha-tubulin are shown. A Wilcoxon test was performed. * $p < 0.05$.

Table S1. Compound descriptions for 27 hit compounds.

Ordered as Fig. 1E

Compound name	Library	Compound description/Therapeutic effect (target and target mechanism)
Triacetylresveratrol	ENZO; Epigenetics	SIRT1 activator
Kenpaullone	ENZO; Kinase Inhibitor	GSK-3β inhibitor
AG-1295	ENZO; Kinase Inhibitor	Tyrosine Kinase inhibitor
Y-27632.2HCl	ENZO; Kinase Inhibitor	Rho Kinase inhibitor
Resveratrol	ENZO; Epigenetics	SIRT1 activator
Apigenin	ENZO; Kinase Inhibitor	CK-II inhibitor
PD-98059	ENZO; Kinase Inhibitor	MEK inhibitor
GF 109203X	ENZO; Kinase Inhibitor	PKC inhibitor
PP1	ENZO; Kinase Inhibitor	Src inhibitor
AG-1296	ENZO; Kinase Inhibitor	PDGFRK
Indirubin-3'-monoxime	ENZO; Kinase Inhibitor	GSK-3β inhibitor
Genistein	ENZO; Kinase Inhibitor	Tyrosine Kinase inhibitor
Dyclonine hydrochloride	Prestwick Chemical	Local anesthetic<br>(Voltage-gated NA+ channel inhibitor, Aldehyde dehydrogenase inhibitor)
Chlorothiazide	Prestwick Chemical	Antihypertensive, Diuretic<br>(Thiazide-sensitive sodium-chloride cotransporter inhibitor, Carbonic anhydrase I, II, IV inhibitor)
Pinacidil	Prestwick Chemical	Antihypertensive, Vasodilator, Anti-inflammatory<br>(K+ channel Ca2+ dependant activator)
Leflunomide	Prestwick Chemical	Immunosuppressant, Antineoplastic<br>(Dihydroorotate dehydrogenase inhibitor)
Rabeprazole sodium salt	Prestwick Chemical	Antiulcer<br>(H+/K+ ATPase)
Ceftazidime pentahydrate	Prestwick Chemical	Antibacterial<br>(Penicillin-binding protein 1A, 1B, 2,3,4 inhibitor)
Phenazopyridine hydrochloride	Prestwick Chemical	Analgesic, Antidotes<br>(microtubule associated protein tau inhibitor, estrogen receptor 1 agonist, nuclear receptor 1H4 antagonist)
Abacavir Sulfate	Prestwick Chemical	Antiviral<br>(Reverse transcriptase inhibitor)
Nabumetone	Prestwick Chemical	Analgesic, Anti-inflammatory<br>(Cyclooxygenase)
Metolazone	Prestwick Chemical	Antihypertensive, Diuretic, Antineoplastic<br>(Thiazide-sensitive sodium-chloride cotransporter inhibitor)
Quetiapine hemifumarate	Prestwick Chemical	Antipsychotic<br>(Dopaminergic and 5-HT receptors antagonist)
Oxfendazole	Prestwick Chemical	Antihelmitic
Ethinylestradiol	Prestwick Chemical	Contraceptive<br>(Estrogen receptor)
Luteolin	Prestwick Chemical	Expectorant, Antineoplastic
Ezetimibe	Prestwick Chemical	Hypocholesterolemic<br>(Niemann-Pick C1-like protein 1)

Table S2. Z Scores for all screened compounds.

Available for download at
https://journals.biologists.com/jcs/article-lookup/doi/10.1242/jcs.263487#supplementary-data

Journal of Cell Science • Supplementary information

Table S3. Compounds used for validation of screening results.

Compound	Supplier(s)	Catalogue number
Resveratrol	ENZO	BML-FR104-0100
Triacetylresveratrol	ENZO	BML-FR119-0010
Apigenin	ENZO	BML-EI345-0020
Kenpaullone	ENZO<br>Cayman Chemical	BML-EI310-0005<br>10010239
Indirubin-3'-monoxime	ENZO	BML-CC207-0001
PD-98059	ENZO	BML-EI360-0005
AG-1296	ENZO	BML-EI303-0005
GF 109203X	ENZO	BML-EI246-0001
TYRPHOSTIN AG 1295	ENZO	ALX-270-035-M001
Genistein	ENZO	ALX-350-006-M010
Hypericin	ENZO	ALX-350-030-M001
Phenazopyridine (hydrochloride)	Cayman Chemical	29683
Nabumetone	Cayman Chemical	20251
Rabeprazole (sodium salt)	Cayman Chemical	14939
Dyclonine (hydrochloride)	Cayman Chemical	27667
Luteolin	Cayman Chemical	10004161
Metolazone	Cayman Chemical	15987
Leflunomide	Cayman Chemical	14860
Quetiapine (hemifumarate)	Cayman Chemical	14152
Chlorothiazide	Cayman Chemical	17909
Dichlorphenamide	Cayman Chemical	23658
Oxfendazole	LKT Laboratories	09322
Ezetimibe	Adooq Bioscience	A10379
Ethinylestradiol	Sigma	E4876
Pinacidil monohydrate	Santa Cruz Biotechnology	SC-203198
BIO	Cayman Chemical	13123
1-Azakenpaullone	Cayman Chemical	16733

Journal of Cell Science • Supplementary information

Table S4. Donor information for primary human airway basal cell cultures.

Donor ID	Type	Location	Self-reported Ethnicity	Age at collection (yrs)	Sex	Smoking status	Respiratory comorbidities recorded
**1**	Bronchial	Lingula	White	73	M	Former	No
**2**	Bronchial	2a: RUL<br>2b: RML	White	2a: 77<br>2b: 80	M	Former	Dysplasia and emphysema
**3**	Bronchial	LLL	White	71	M	Current	No
**4**	Bronchial	LLL	Black African	61	M	Never	Asthma
**5**	Bronchial	LUL/LLL carina	White	67	M	Current	No
**6**	Bronchial	LUL	White	58	F	Current	Previous squamous cell cancer
**7**	Bronchial	Unknown	White	63	M	Never	No
**8**	Bronchial	Unknown	Black African	60	M	Current	No
**9**	Lobectomy	RUL	White	76	M	Former	COPD and lung emphysema
**10**	Lobectomy	LLL	White	78	F	Former	Previous small cell cancer
**11**	Lobectomy	LLL	White	73	F	Former	No
**12**	Bronchial	RUL	White	73	M	Current	No

Journal of Cell Science • Supplementary information

Table S5. qPCR primer sequences.

Gene	Species	Primer sequence (5'-3')
LEF1	human	Forward: AGAACACCCCGATGACGGA<br>Reverse: GGCATCATTATGTACCCGGAAT
AXIN2	human	Forward: CAACACCAGGCGGAACGAA<br>Reverse: GCCCAATAAGGAGTGTAAGGACT
CCND1	human	Forward: CCGAGAAGCTGTGCATCTACAC<br>Reverse: AGGTTCCACTTGAGCTTGTTCAC
MYC	human	Forward: CCTGGTGCTCCATGAGGAGAC<br>Reverse: CAGACTCTGACCTTTTGCCAGG
RPS13	human	Forward: TCGGCTTTACCCTATCGACGCAG<br>Reverse: ACGTACTTGTGCAACACCATGTGA
GAPDH	human	Forward: TGATGACATCAAGAAGGTGGTGAAG<br>Reverse: TCCTTGGAGGCCATGTAGGCCAT
Lef1	mouse	Forward: TGTTTATCCCATCACGGGTGG<br>Reverse: CATGGAAGTGTCGCCTGACAG
Axin2	mouse	Forward: TGACTCTCCTTCCAGATCCCA<br>Reverse: TGCCCACACTAGGCTGACA
Ccnd1	mouse	Forward: GCAGAAGGAGATTGTGCCATCC<br>Reverse: AGGAAGCGGTCCAGGTAGTTCA
Myc	mouse	Forward: CAGAGGAGGAACGAGCTGAAGCGC<br>Reverse: TTATGCACCAGAGTTTCGAAGCTGTTCG
Actb	mouse	Forward: ATGGCTGGGGTGTTGAAGGT<br>Reverse: ATCTGGCACCACACCTTCTACAA
Hprt1	mouse	Forward: CTGGTGAAAAGGACCTCTCGAAG<br>Reverse: CCAGTTTCACTAATGACACAAACG

Journal of Cell Science • Supplementary information

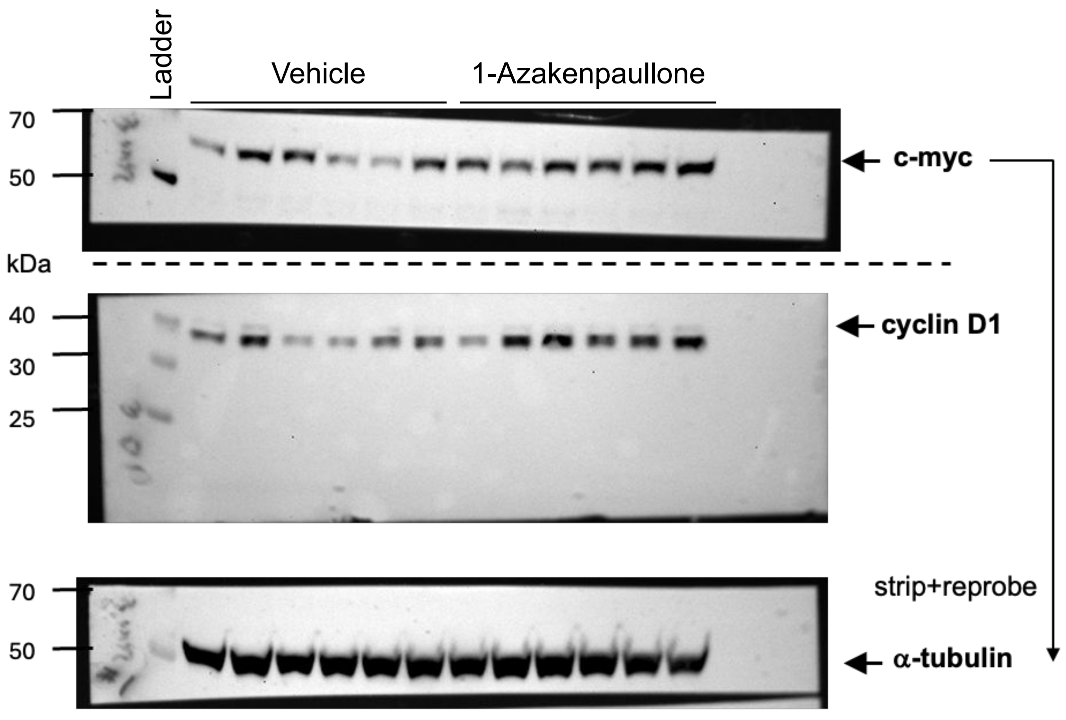

Fig. S4. Blot transparency. Uncropped scans of Fig. S3C Western blots. Dashed line indicates where membrane was cut. The membrane used for c-myc was stripped and reprobed with anti- alpha-tubulin antibody as a loading control.
