# Supplementary material for: Compound screening in human airway basal cells identifies Wnt pathway activators as potential pro-regenerative therapies
Source: J Cell Sci. 2025 Apr 14;138(7):jcs263487. doi: 10.1242/jcs.263487 (PMC12045047; doi:10.1242/jcs.263487)
Supplement: Supplementary file 1 [file joces-138-263487_review_history.pdf]

Compound screening in human airway basal cells identifies Wnt pathway activators as potential pro-regenerative therapies

Yuki Ishii, Jessica C. Orr, Marie-Belle El Mdawar, Denise R. Bairros de Pilger, David R. Pearce, Kyren A. Lazarus, Rebecca E. Graham, Marko Z. Nikolić, Robin Ketteler, Neil O. Carragher, Sam M. Janes and Robert E. Hynds
DOI: 10.1242/jcs.263487

Editor: Simon Cook

Review timeline

Original submission:	13 August 2024
Editorial decision:	29 October 2024
First revision received:	10 February 2025
Accepted:	4 March 2025

Original submission

<u>First decision letter</u>

MS ID#: jcs.263487

MS TITLE: Compound screening in primary human airway basal cells identifies Wnt pathway activators as potential pro-regenerative therapies

AUTHORS: Yuki Ishii; Jessica C Orr; Marie-Belle El Mdawar; Denise R Bairros De Pilger; David R Pearce; Kyren A Lazarus; Rebecca E Graham; Marko Z Nikolic; Robin Ketteler; Neil O Carragher; Sam M Janes; Robert E Hynds

ARTICLE TYPE: Short Report

Dear Dr Hynds,

We have now reached a decision on the above manuscript. I apologise that this has taken longer than i would like; it has proven difficult to find reviewers who would accept our invitation.

To see the reviewers' reports and a copy of this decision letter, please go to:

As you will see, the reviewers raise a number of criticisms that prevent me from accepting the paper at this stage. They suggest, however, that a revised version might prove acceptable, if you can address their concerns. If you think that you can deal satisfactorily with the criticisms on revision, I would be pleased to see a revised manuscript. We would then return it to the reviewers.

SUMMARY OF THE ADVANCE MADE IN THIS PAPER AND ITS POTENTIAL SIGNIFICANCE TO THE FIELD

The failure to effectively repair epithelial barriers following injury and infection can lead to debilitating disease. Consequently, promoting airway epithelial regeneration has the potential to treat a host of respiratory disorders such as asthma, tracheobronchitis and cytonecrosis. The identification of small molecules that could be beneficial requires the ability to screen in a high

© 2025. Published by The Company of Biologists under the terms of the Creative Commons Attribution License (https://creativecommons.org/licenses/by/4.0/).

throughout and reproducible manner, and preferentially using primary cells to more closely mimic what is likely to occur in vivo. The authors establish such a screen using 2D and 3D primary cell cultures in combination with a pHIV-Luc-zsGreen lentivirus to enable luciferase monitoring of cell proliferation in response to treatment with small molecules. The authors validate the set up for the screen with a small molecule, Y-27632, that is known to induce basal cell proliferation. They then use their screen to look for novel compounds that could boost cell proliferation using compounds that are approved for clinical use by the FDA, EMA and other agencies. The authors then validate several positive hits from the screen, focusing in more detail on molecules that have been implicated in regulating Wnt signalling. They then validate that these molecules activate Wnt signalling and cell proliferation both in 2D and 3D culture and that one of the molecules boosts Wnt signalling and proliferation in the mouse lung when delivered by intraperitoneal injection. All the experiments are well controlled and the use of a Wnt inhibitor in combination with the small molecules demonstrates cell proliferation is driven by Wnt signalling. The compounds themselves are not particularly novel since they have been shown to boost Wnt signalling previously. Nor is the finding that Wnt signalling acts as a pro-proliferative signal in airway epithelial cells. However, the screen is a nice proof of principle and the screen could be used on an expanded number of compounds in future to find novel hits.

SUGGESTIONS TO AUTHORS

Page 5… line 19… it would be good to indicate in the main text what Y-27632 inhibits and its mechanism of action in boosting proliferation since it is the positive control the screen is validated on.

Page 6… line 23… references required for the following statement. "Four validated hit compounds - indirubin-3'-monoxime, kenpaullone, nabumetone and phenazopyridine - have been previously reported to activate canonical Wnt signalling." It would also help the reader to explain any understood mechanism of action for these compounds in modulating Wnt.

Figure 2B… kenpaullone is stated as increasing colony forming units but this only true of the lower doses with 5uM decreasing efficiency. Yet 5uM robustly elevates organoid area in the subsequent assay. Do the authors have an explanation for why the high dose inhibits colony forming but yet is the best concentration for increasing organoid size?

Figure 2D and 3G would benefit from a cropped and magnified insert as it is hard to see the markers at the current magnification.

SUMMARY OF THE ADVANCE MADE IN THIS PAPER AND ITS POTENTIAL SIGNIFICANCE TO THE FIELD

The study by Ishii and colleagues addresses whether they promote the regenerative programme of lung airway basal cells using repurposed drugs. The authors carry out a small molecule screen to identify compounds that promote the proliferation of primary human basal cells. Of the 16 hits identified in the screen, the authors focus on the 4 compounds that have previously been shown to promote Wnt pathway activity. Likely, many of the 16 compounds were GSK3 inhibitors, and an interesting addition to the study would have been to confirm this.
Of the 4 hits, the GSK3 inhibitor 1-azakenpaullone was taken forward for in vivo studies. It is not clear why 1-azakenpaullone was taken forward (other than it has previously been administered to mice), as the hit Kenpaullone had the largest in vitro effect on bronchial cell proliferation and impacted Wnt pathway activity. Moreover, if the authors believe that the proliferative effects of drug treatment were mediated directly through GSK3 inhibition, there are better, more specific clinically-used alternatives to 1-azakenpaullone for the in vivo study.

SUGGESTIONS TO AUTHORS

© 2025. Published by The Company of Biologists under the terms of the Creative Commons Attribution License (https://creativecommons.org/licenses/by/4.0/).

The overall novelty of the study is a validated screening platform for identifying small molecules that promote lung airway basal cell proliferation and perhaps the use of the screen to confirm that the Wnt pathway activity is a driver of proliferation of lung airway basal cells. The study could be sufficient for publication in JCS, if the points above and the following specific points are addressed:

(1) In order to establish the robustness of the screen, the secondary screen confirming the hits (Fig. 2A and Suppl. Fig 2A) should be carried out using a method different from luciferase activity, for instance the cell titerglo assay that was used in subsequent experiments.

(2) The ROCKi compound, in general, increases survival. The colony formation assay (Fig. 2B) measures survival (and proliferation to some degree if one considers colony size). Is there any way to dissect out the consequences of the hit compounds on survival and proliferation? For instance, are there differences in the size of the colonies?

(3) It is not clear whether the organoid assays in Fig. 2C and Fig. S2B display biologically significant differences. Is the idea that there are 60 points per drug treatment that are composed of 12 replicate wells (for 5 donors), all compared to a single control well? If so, wouldn't the replicate wells qualify as technical replicates and there should be 5 data points per well reflecting the 5 donors? Please correct where I am wrong.

(4) Can the authors provide the reference(s) for this statement: "Four validated hit compounds - indirubin-3'-monoxime, kenpaullone, nabumetone and phenazopyridine - have been previously reported to activate canonical Wnt signalling."

(5) A direct experiment should be carried to determine whether all of the hits from the screen target GSK3 activity and impact basal cell proliferation.
Moreover, do Wnt ligands (for instance the canonical pathway agonist Wnt3A) or other GSK3 inhibitors (chiron, lithium) promote basal cell proliferation?

(6) With regards to the in vivo experiments with 1-Azakenpaullone: is the idea that the $p<0.015$ level of significance for Edu positive cells in trachea, and not lung, indicative of a difference in response to treatment with the GSK3 inhibitor? These don't seem to mesh with the tracheal and lung qRT-PCR and western blots - myc expression goes up significantly in the lung but not trachea and protein expression of myc and Ccnd1 goes up in lung (what about trachea?). Are values significantly different in order to delineate differences in expression? Are changes in Myc expression a surrogate for Wnt pathway activity or something else? Finally, it should be justified why a Wilcoxon test is being used here when the data seems to require a parametric test.

First revision

<u>Author response to reviewers' comments</u>

We thank the reviewers for taking the time to provide valuable feedback on our manuscript. We address their comments (**shown in bold**) point-by-point below.

Reviewer 1:

SUMMARY OF THE ADVANCE MADE IN THIS PAPER AND ITS POTENTIAL SIGNIFICANCE TO THE FIELD

The failure to effectively repair epithelial barriers following injury and infection can lead to debilitating disease. Consequently, promoting airway epithelial regeneration has the potential to treat a host of respiratory disorders such as asthma, tracheobronchitis and cytonecrosis. The identification of small molecules that could be beneficial requires the ability to screen in a high throughput and reproducible manner, and preferentially using primary cells to more closely

© 2025. Published by The Company of Biologists under the terms of the Creative Commons Attribution License (https://creativecommons.org/licenses/by/4.0/).

mimic what is likely to occur in vivo. The authors establish such a screen using 2D and 3D primary cell cultures in combination with a pHIV-Luc-zsGreen lentivirus to enable luciferase monitoring of cell proliferation in response to treatment with small molecules. The authors validate the set up for the screen with a small molecule, Y-27632, that is known to induce basal cell proliferation. They then use their screen to look for novel compounds that could boost cell proliferation using compounds that are approved for clinical use by the FDA, EMA and other agencies. The authors then validate several positive hits from the screen, focusing in more detail on molecules that have been implicated in regulating Wnt signalling. They then validate that these molecules activate Wnt signalling and cell proliferation both in 2D and 3D culture and that one of the molecules boosts Wnt signalling and proliferation in the mouse lung when delivered by intraperitoneal injection. All the experiments are well controlled and the use of a Wnt inhibitor in combination with the small molecules demonstrates cell proliferation is driven by Wnt signalling. The compounds themselves are not particularly novel since they have been shown to boost Wnt signalling previously. Nor is the finding that Wnt signalling acts as a pro-proliferative signal in airway epithelial cells. However, the screen is a nice proof of principle and the screen could be used on an expanded number of compounds in future to find novel hits.
We thank the reviewer for their summary - this is a fair assessment of the advances in our manuscript.

SUGGESTIONS TO AUTHORS

Page 5… line 19… it would be good to indicate in the main text what Y-27632 inhibits and its mechanism of action in boosting proliferation since it is the positive control the screen is validated on.
We have modified the text, which now clarifies this as follows:
"As a positive control, we tested Y-27632, a small-molecule inhibitor of Rho-associated protein kinases (ROCK1/2) (Davies et al., 2000) that is known to induce basal cell proliferation (Horani et al., 2013; Reynolds et al., 2016; Witkowski et al., 2023)."
Page 6… line 23… references required for the following statement. "Four validated hit compounds - indirubin-3'-monoxime, kenpaullone, nabumetone and phenazopyridine - have been previously reported to activate canonical Wnt signalling." It would also help the reader to explain any understood mechanism of action for these compounds in modulating Wnt.
We have explained the mechanisms of action of the hit compounds as follows:
"Five validated hit compounds - indirubin-3'-monoxime, kenpaullone, nabumetone, phenazopyridine and luteolin - have been previously associated with canonical Wnt signalling. Indirubins and kenpaullones inhibit GSK3β through competitive binding with ATP at the catalytic site (Leclerc et al., 2001; Zaharevitz et al., 1999). Nabumetone (a non-steroidal anti-inflammatory drug) and phenazopyridine (a urinary tract analgesic) were identified as Wnt pathway activators in a luciferase reporter cell-based screen (Costa et al., 2021), although the mechanism of pathway activation is less clear for these molecules. Luteolin's role in Wnt signalling is context-dependent, upregulating beta catenin in periodontal ligament cells (Quan et al., 2019), but downregulating pathway activation in prostate cancer cell lines (Han et al., 2018)."
Additionally, we have now shown experimentally that these compounds activate the TOPFlash reporter in HBEC3-KT cells in our hands (Fig. 3A).
Figure 2B… kenpaullone is stated as increasing colony forming units but this is only true of the lower doses with 5uM decreasing efficiency. Yet 5uM robustly elevates organoid area in the subsequent assay. Do the authors have an explanation for why the high dose inhibits colony forming but yet is the best concentration for increasing organoid size?
We think that this difference might be explained by technical aspects of the two assays. In colony formation assays, the cells are in 2D, on 3T3-J2 feeder cells, and on a stiff plastic substrate, while in the bronchosphere/organoid assays they are encapsulated in 5% Matrigel on a bed of 25% Matrigel, so some differences in proliferative responses might be expected. Moreover, we must be cautious about direct comparisons of the concentrations of drug between these assays because, as per the materials and methods, cells in the colony forming assay experience 5 μM kenpaullone at all medium changes, whereas medium containing 5 μM kenpaullone is added at days 3, 10 and 17 in the

© 2025. Published by The Company of Biologists under the terms of the Creative Commons Attribution License (https://creativecommons.org/licenses/by/4.0/).

organoid/bronchosphere assay meaning that they experience a lower concentration than that at each feed.

Figure 2D and 3G would benefit from a cropped and magnified insert as it is hard to see the markers at the current magnification.
Agreed - in the revision we show higher magnification images.
Reviewer 2: SUMMARY OF THE ADVANCE MADE IN THIS PAPER AND ITS POTENTIAL SIGNIFICANCE TO THE FIELD
The study by Ishii and colleagues addresses whether they promote the regenerative programme of lung airway basal cells using repurposed drugs. The authors carry out a small molecule screen to identify compounds that promote the proliferation of primary human basal cells. Of the 16 hits identified in the screen, the authors focus on the 4 compounds that have previously been shown to promote Wnt pathway activity. Likely, many of the 16 compounds were GSK3 inhibitors, and an interesting addition to the study would have been to confirm this.
Thanks for this suggestion. In the revised manuscript, we have now assessed the 17 hits in the TOPFlash assay in HBEC3-KT cells (Fig. 3A). The five Wnt activating compound hits from screening activated Wnt signalling to the greatest extent and, as the reviewer predicted, some of the other compounds showed low levels of Wnt pathway activation (Fig. 3A).

Of the 4 hits, the GSK3 inhibitor 1-azakenpaullone was taken forward for in vivo studies. It is not clear why 1-azakenpaullone was taken forward (other than it has previously been administered to mice), as the hit Kenpaullone had the largest in vitro effect on bronchial cell proliferation and impacted Wnt pathway activity. Moreover, if the authors believe that the proliferative effects of drug treatment were mediated directly through GSK3 inhibition, there are better, more specific clinically-used alternatives to 1-azakenpaullone for the in vivo study.
We clarify this in the revised manuscript as follows:
"Kenpaullone, indirubin and their derivatives 1-azakenpaullone and BIO consistently promoted basal cell proliferation in our *in vitro* studies with the latter two being most effective. While BIO is a potent GSK3β inhibitor, it also targets CDKs (Meijer et al., 2003). Given that 1-azakenpaullone has a greater selectivity for GSK3β (Kunick et al., 2004) and it demonstrated the strongest enhancement of cell proliferation using a 3D organoid model, we assessed the potential of 1-azakenpaullone to modulate airway basal cell proliferation and Wnt pathway activation *in vivo*."
SUGGESTIONS TO AUTHORS
The overall novelty of the study is a validated screening platform for identifying small molecules that promote lung airway basal cell proliferation and perhaps the use of the screen to confirm that the Wnt pathway activity is a driver of proliferation of lung airway basal cells. The study could be sufficient for publication in JCS, if the points above and the following specific points are addressed:
(1) In order to establish the robustness of the screen, the secondary screen confirming the hits (Fig. 2A and Suppl. Fig 2A) should be carried out using a method different from luciferase activity, for instance the cell titerglo assay that was used in subsequent experiments.
We demonstrate that luciferase transduction of airway basal cells (as deployed in the screen) correlated with ground truth DAPI nuclei counts (Fig. 1B, S1D). We also demonstrate a concentration-response for the hit compounds from the ENZO compound library using CellTiter-Glo rather than transduction with luciferase (Fig. S2B). The observed responses were well-correlated with the screen data. In addition, we have performed an additional CellTiter-Glo experiment in HBEC3-KT cells, which further confirmed that all hit compounds from the screen increased basal cell proliferation (Fig. 3A), reinforcing the robustness of the screen.
(2) The ROCKi compound, in general, increases survival. The colony formation assay (Fig. 2B) measures survival (and proliferation to some degree if one considers colony size). Is there any way to dissect out the consequences of the hit compounds on survival and proliferation? For instance, are there differences in the size of the colonies?
To dissociate the effects of compounds on survival versus proliferation, we plated cells in the control medium and added hit compounds the following day. Thus, the colony formation assays in our study only assess the effect of compounds on the proliferation of adherent epithelial cells.

© 2025. Published by The Company of Biologists under the terms of the Creative Commons Attribution License (https://creativecommons.org/licenses/by/4.0/).

(3) It is not clear whether the organoid assays in Fig. 2C and Fig. S2B display biologically significant differences. Is the idea that there are 60 points per drug treatment that are composed of 12 replicate wells (for 5 donors), all compared to a single control well? If so, wouldn't the replicate wells qualify as technical replicates and there should be 5 data points per well reflecting the 5 donors? Please correct where I am wrong.
Thanks for raising this. We agree regarding statistics, and now treat independent donors as experimental replicates. We were keen to maintain a sense of the variability between donors/wells/organoids in the plot but using a violin plot to display organoid-level variability compresses the donor means into the bottom of the y axis and made it difficult to see clear donor-donor variability within the data (see Figure below).

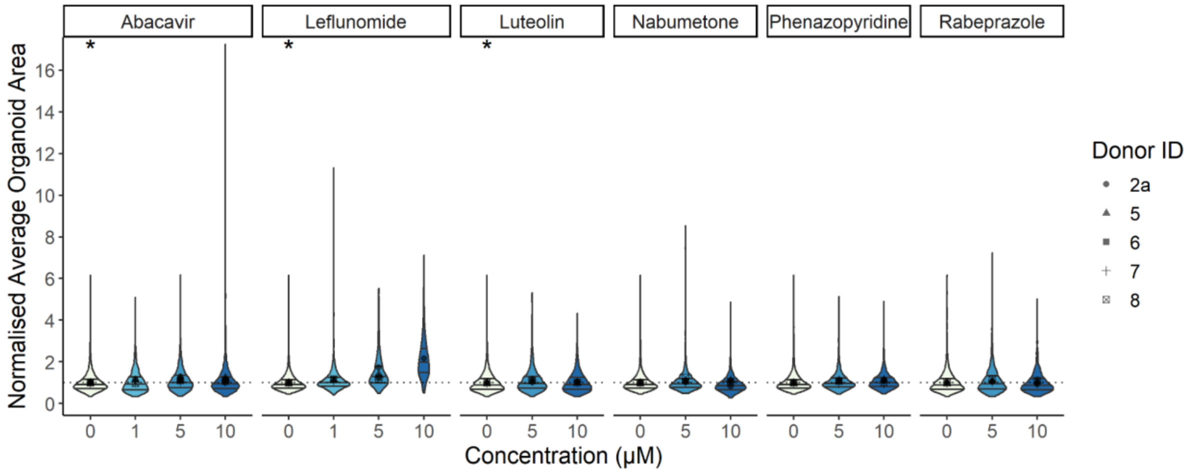

To address this, we now present well-level data points but perform statistics on means per donor for all organoid size experiments.

(4) Can the authors provide the reference(s) for this statement: "Four validated hit compounds - indirubin-3'-monoxime, kenpaullone, nabumetone and phenazopyridine - have been previously reported to activate canonical Wnt signalling."
As per our response to Reviewer 1's second comment, we have now added references to support this and performed TOPFlash for all hits in HBEC3-KT cells (Fig. 3A).

(5) A direct experiment should be carried out to determine whether all of the hits from the screen target GSK3 activity and impact basal cell proliferation.
As per our response to Reviewer 1's second comment, we have now performed an experiment to assess all of the hits in the TOPFlash assay in HBEC3-KT cells (Fig. 3A). As expected, we saw the greatest Wnt pathway activation by compounds previously associated with Wnt pathway activation, and this correlated with increased proliferation.
Moreover, do Wnt ligands (for instance the canonical pathway agonist Wnt3A) or other GSK3 inhibitors (chiron, lithium) promote basal cell proliferation?

In the experiment above, we included lithium as a positive control. As expected, we found that it both activated Wnt signalling and increased cell proliferation (Fig. 3A).

(6) With regards to the in vivo experiments with 1-Azakenpaullone: is the idea that the p<0.015 level of significance for Edu positive cells in trachea, and not lung, indicative of a difference in response to treatment with the GSK3 inhibitor? These don't seem to mesh with the tracheal and lung qRT-PCR and western blots - myc expression goes up significantly in the lung but not trachea and protein expression of myc and Ccnd1 goes up in lung (what about trachea?). Are values significantly different in order to delineate differences in expression? Are changes in Myc expression a surrogate for Wnt pathway activity or something else? Finally, it

© 2025. Published by The Company of Biologists under the terms of the Creative Commons Attribution License (https://creativecommons.org/licenses/by/4.0/).

should be justified why a Wilcoxon test is being used here when the data seems to require a parametric test.
Apologies - the presentation of these data was confusing, with combined tracheal and lung data in both the main figure and the supplementary figure. We analysed the tracheas using EdU staining or qPCR. For the same animals, we took lung tissue and performed additional qPCR and Western blot experiments. In the revised manuscript, we present all tracheal data in the main Fig. 4 and all of the lung data in Fig. S3. In the trachea, the evidence for 1-azakenpaullone action *in vivo* is 1) an increase in proliferation in the trachea and 2) trends towards upregulation of the Wnt target genes *Myc* and *Ccnd1*. The additional data from the lungs further supports bioactivity of the compound *in vivo*, with increased *Myc* expression at the RNA level and increased Myc and cyclin D1 at the protein level.
We performed normality testing per gene using Shapiro-Wilk tests and found that data were not normally distributed. To reflect this, we still use the more conservative non-parametric tests in the revised manuscript.

SUMMARY OF THE ADVANCE MADE IN THIS PAPER AND ITS POTENTIAL SIGNIFICANCE TO THE FIELD

The authors have addressed all of my suggestions.

SUGGESTIONS TO AUTHORS

<u>Second decision letter</u>

MS ID#: jcs.263487R1

MS Title: Compound screening in human airway basal cells identifies Wnt pathway activators as potential pro-regenerative therapies

Authors: Yuki Ishii; Jessica C Orr; Marie-Belle El Mdawar; Denise R Bairros De Pilger; David R Pearce; Kyren A Lazarus; Rebecca E Graham; Marko Z Nikolic; Robin Ketteler; Neil O Carragher; Sam M Janes; Robert E Hynds

Article Type: Short Report

Dear Dr Hynds,

I am happy to tell you that your manuscript has been accepted for publication in Journal of Cell Science, pending standard publication integrity checks.

© 2025. Published by The Company of Biologists under the terms of the Creative Commons Attribution License (https://creativecommons.org/licenses/by/4.0/).
